# A Novel Process for One-Step Separation and Cyclodextrin Inclusion of Ginsenoside Rg5 from Ginseng Stem–Leaf Saponins (GSLS): Preparation, Characterization, and Evaluation of Storage Stability and Bioactivity

**DOI:** 10.3390/foods12122349

**Published:** 2023-06-12

**Authors:** Jianbo Chen, Meijia Li, Xiaohui Huo, Zhiman Li, Di Qu, Jiyue Sha, Yinshi Sun

**Affiliations:** Institute of Special Wild Economic Animals and Plants, Chinese Academy of Agriculture Sciences, Changchun 130112, China; chenjianbo00882@126.com (J.C.);

**Keywords:** ginsenoside Rg5, β-CD, storage stability, solubility, antioxidant activity

## Abstract

Background: Ginsenoside Rg5 has been proven to possess numerous health benefits. However, Rg5 is difficult to prepare using the current methods, and the poor stability and solubility of Rg5 are intractable properties that limit its application. We try to establish and optimize a new method for preparing Rg5. Methods: Different amino acids acted as catalysts, and reaction conditions were investigated to transform Rg5 in GSLS. Different CDs and reaction conditions were investigated for the preparation of CD-Rg5 based on yield and purity; ESI-MS, FT-IR, XRD and SEM analyses were used to prove the formation of the CD-Rg5 inclusion complex. Both the stability and bioactivity of β-CD-Rg5 were investigated. Results: The content of Rg5 reached 140.8 mg/g after transformation of GSLS using Asp as a catalyst. The yield of β-CD-Rg5 reached a maximum of 12% and a purity of 92.5%. The results showed that the β-CD-Rg5 inclusion complex can improve its stability of Rg5 against light and temperature. Antioxidant activity analyses against DPPH, ABTS^+^, and Fe^2+^ chelation showed enhanced antioxidant activity of the β-CD-Rg5 inclusion complex. Conclusions: A novel and effective strategy for the separation of Rg5 from ginseng stem–leaf saponins (GSLS) was developed to improve the stability, solubility, and bioactivity of Rg5.

## 1. Introduction

Ginseng is used as a medicine that is beneficial for nutrition and health in East Asia, North America, and Europe [1,2]. As the main active ingredient of ginseng, ginsenoside has become a popular food functional and source of health-promoting molecules [3,4]. Ginsenoside Rg5 (Rg5) has been demonstrated to have abundant biomedical activities, including anticancer, anti-allergy, antimicrobial, antidepression, and anti-inflammatory properties, as well as improved memory and whitening [5,6,7,8]. Unfortunately, Rg5 is very unstable, has low solubility in water, and degrades under environmental stimuli such as light, heat, and oxygen, which reduces its biological activity and lowers its potency as a functional ingredient [9].

Rg5 is present at very low levels in natural ginseng plants, with a mass of approximately 0.03–0.08% in white ginseng [10]. Rg5 is difficult to prepare using the current methods of extraction, separation, and purification for Rg5. The isolation and purification methods of Rg5 include preparative HPLC, silica gel column chromatography, and macroporous resin [11]. The disadvantages of these methods include long separation times, high cost, environmentally unfriendly processes, and unsuitability for industrial production [9,12].

At present, Rg5 is generally extracted from red ginseng and black ginseng [9]. Studies have shown that ginseng stem–leaf saponins (GSLS) are similar to those in ginseng roots, and the total saponins in ginseng leaves are higher than those in ginseng roots [13,14]. After the harvest of ginseng, the stems and leaves are often abandoned. Poor utilization not only wastes resources but also causes environmental pollution [15,16]. The stem and leaf are rich sources of ginseng that can be harvested every year, and the raw material is inexpensive. Our latest research has reported a method of transforming common ginsenosides into Rk1/Rg5 in American ginseng [17]. Therefore, using special transformation technology, GSLS could become one of the richest sources of Rg5.

Cyclodextrin (CD) is a cyclic, natural, or semiartificial oligosaccharide that generally contains 6 (α-CD), 7 (β-CD) or 8 (γ-CD) glucopyranose units. A broad host–guest inclusion complex can be formed because of its unique structure that includes a hydrophilic edge and a lipophilic interior [18,19]. As a new type of biological agent, β-CD can be combined with active substances to improve their stability and bioavailability; thus, it is widely used in the food industry [20]. In addition, in the aqueous solution, β-CD provides more effective exploitation of natural plant resources, which interact with target molecules (bioactive substances) through noncovalent bonds [21]. Compared with other chemical extraction methods, CDs have lower biological toxicity and produce less secondary pollution. In brief, CD is an environmentally friendly additive that can rapidly and efficiently extract hydrophilic and hydrophobic compounds from plants.

Based on these properties, we attempted to prepare Rg5 using CDs from GSLS. During the experiment, we found that CDs could selectively incorporate Rg5 under acidic conditions, and the inclusion complex of Rg5 was then precipitated from the acidic solution. Based on this particular phenomenon, the primary objective of this study was to optimize methods of separating Rg5 from GSLS with CDs. A secondary goal was to confirm the inclusion complex. Samples of the theoretically formed inclusion compounds were subjected to electrospray ionization mass spectrometry (ESI-MS), Fourier-transform infrared (FT-IR) spectroscopy, X-ray diffraction (XRD) spectroscopy, thermogravimetric analysis (TGA), and scanning electron microscopy (SEM). A third goal was to investigate the storage stability and bioactivity of the resulting product.

## 2. Materials and Methods

### 2.1. Materials

GSLS was bought from Andong Biotechnology Co., Ltd. (Changchun, China). Rg5, Asp, Glu, Arg, His, Lys, and Lys were bought from Yuanye Biotechnology Co., Ltd. (Shanghai, China). α-CD, β-CD, γ-CD, methyl-β-CD, HP-β-CD, and glucose-β-CD were purchased from Shandong Xinda Fine Chemical Company (Jinan, China). Alcohol for analysis was purchased from Shanghai Chemical Industry (Shanghai, China).

### 2.2. Rg5 Conversion in GSLS

In total, 5 g of amino acid was added to 100 mL of water and stirred, while being heating to 110 °C to dissolve the amino acids; 10 g of GSLS was then added to the amino acid solution and stirred for 1 h. Two acidic amino acids (Asp and Glu) and three basic amino acids (Arg, His and Lys) were used for amino acid screening. The reaction mixture was filtered through a 0.22 micron filter. Rg5 content was determined by UPLC (Waters, Manchester, UK). The operation was performed three times. Four factors affecting the conversion rate of Rg5 were then investigated: 70–120 °C for reaction temperature; 1–14% for amino acid concentration; 5–40 mL/g for liquid-solid ratio; and 0.5–3 h for reaction time.

### 2.3. Preparation of the β-CD-Rg5 Inclusion Complex from GSLS

**Figure 1:**
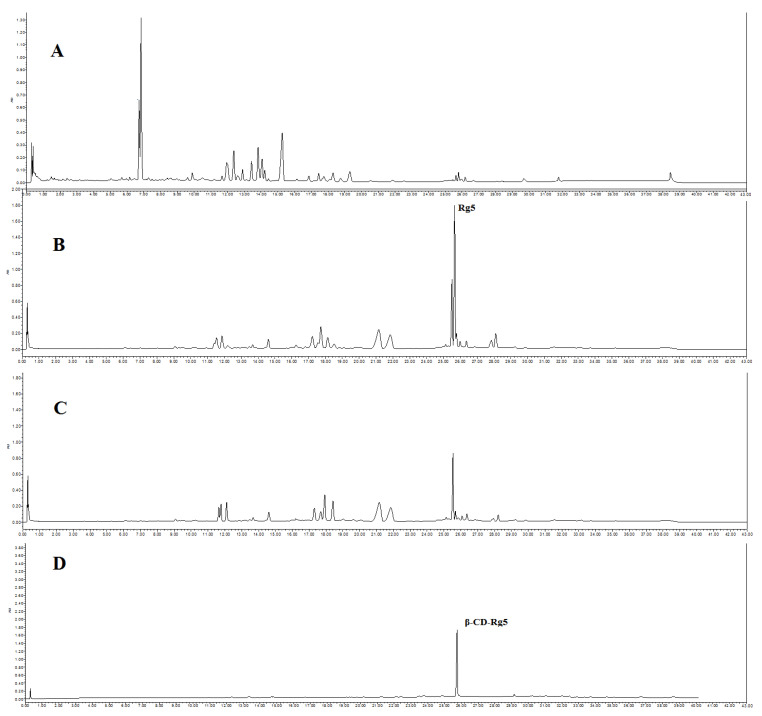
UPLC spectrum of (**A**) GLSL; (**B**) GSLS after amide acid conversion; (**C**) after GSLS with β-CD inclusion; (**D**) β-CD-Rg5 inclusion complex.

Six CDs, including α-CD, β-CD, γ-CD, methyl-β-CD, HP-β-CD, and glucose-β-CD, were chosen. Conversion liquid mixtures of GSLS and CDs were mixed at a CDs and GSLS ratio of 1.0 and stirred at 30 °C. After standing for 6 h, the precipitate was obtained via centrifugation, and the crude CD-Rg5 inclusion complex was also obtained. The refined CD-Rg5 inclusion complex was obtained by washing twice with 10% amino acid aqueous solution and twice with 5% ethanol. UPLC was used to confirm that the precipitate was a β-CD-Rg5 inclusion complex (Figure 1). The four factors affecting the yield and purity of Rg5, including reaction temperature (15–40 °C), β-CD and GSLS ratio (0.2–1.4), pH value (1–7), and reaction time (1–6 h), were investigated.

### 2.4. Characterization of β-CD-Rg5

#### 2.4.1. Electrospray Ionization Mass Spectrometry (ESI-MS)

Rg5, the β-CD-Rg5 inclusion complex, and the β-CD/Rg5 physical mixture were analyzed with an ABIQ-Trap mass spectrometer (Foster City, CA, USA) and an ABI Q-TOF-MS (Foster City, CA, USA). The devices were equipped with EI source and interfaced with software running ABA (version 1.4). 

#### 2.4.2. Fourier-Transform Infrared (FTIR) Spectroscopy

FTIR was recorded using a Spectrum Two L1600300 spectrometer (Perkin Elmer, Waltham, MA, USA). Any possible β-CD-Rg5 interactions were analyzed by FTIR spectroscopy (Bruker, Germany). Rg5, β-CD, the β-CD-Rg5 inclusion complex and the β-CD/Rg5 physical mixture were inserted into KBr microparticles at 1:10 KBr/sample. The wavenumber of the scan was 4000–400 cm^−1^ [22].

#### 2.4.3. X-ray Diffraction Analysis

The crystalline structure of Rg5, β-CD, the β-CD-Rg5 inclusion complex and the β-CD/Rg5 physical mixture was assessed using a commercial X-ray diffractometer (Bruker, Germany), a 40 kV voltage, and a range of diffraction angles from 4 to 40° in 2θ.

#### 2.4.4. Thermogravimetric Analysis (TGA)

Rg5, β-CD, the β-CD-Rg5 inclusion complex, and the β-CD/Rg5 physical mixture were assessed using the thermogravimetric method (USA, SDT 650, TA). Each sample was scanned between 50 °C and 950 °C at a constant speed with a temperature of 10 °C/min. All assays were performed in a nitrogen environment. The weight loss was calculated as the ratio of the actual weight during the heating period to the initial mass of the samples.

#### 2.4.5. Scanning Electron Microscopy (SEM)

Rg5, β-CD, the β-CD-Rg5 inclusion complex and the β-CD/Rg5 physical mixtures were observed using SEM (Salem, OR, USA). All samples were sprayed with gold–palladium alloy (Watford, UK). The scan image was captured with an acceleration voltage of 20 kV at a magnification of 2000× (10 microns).

### 2.5. Stability of β-CD-Rg5

The effects of light on the stability of Rg5, β-CD, the inclusion complexes of β-CD-Rg5 and the β-CD/Rg5 physical mixtures were studied. The samples were placed under a color temperature incandescent lamp with a 5000 k electronically ballasted light source for 30 d. For the effect of temperature on stability, Rg5, β-CD, β-CD-Rg5, and the β-CD/Rg5 physical mixtures were heated at 45 °C for 30 d. The samples were placed at room temperature, and their content was measured by HPLC.

### 2.6. Dissolution In Vitro

A known amount of Rg5, β-CD, the β-CD-Rg5 inclusion complex, and the β-CD/Rg5 physical mixture was placed in a small cup with distilled water. The volume was 200 mL, the speed was 200 rpm/min, and the water bath temperature was 37 ± 0.5 °C. The content of Rg5 was determined using UPLC, and the cumulative dissolution rate was calculated by adding 2.0 mL isothermal medium.

### 2.7. Antioxidant Activity

#### 2.7.1. DPPH Free Radical Scavenging Ability

The scavenging effects of Rg5, β-CD, β-CD-Rg5, and the β-CD/Rg5 physical mixtures on DPPH were studied. The process was previously reported by Andrade et al. [23]. Briefly, 5 different concentrations (0.2, 0.4, 0.6, 0.8, and 1.0 mg/mL) were prepared. Next, 1 mL of the prepared sample was mixed with 2 mL of DPPH (0.01 mg/mL) in absolute ethanol (test group). Next, 1.0 mL of the test sample (control) was added to 2.0 mL of absolute ethanol. DPPH absolute ethanol (1.0 mL) was added to 1 mL deionized water. The three mixtures were shaken well, and keep them at room temperature for 60 min. The absorbance was then measured at 517 nm. Rg5, β-CD, β-CD-Rg5, and the physical mixtures used a similar approach. The reference standard was Vitamin C. The following formula was used:

$$DPPH\;radical\;scavenging\;activity\;(\%)\;=\;1-\frac{A0-A1}{A2}\times 100$$

where A0 is the absorption of the test group, A1 is the absorption of the control group, and A2 is the absorption of the blank group. 

#### 2.7.2. ABTS^+^ Free Radical Scavenging Ability

The scavenging ability of Rg5, β-CD, the β-CD-Rg5 inclusion complex, and the β-CD/Rg5 physical mixture against ABTS^+^ was carried out as described previously by Aarland et al. [24]. Briefly, 0.2, 0.4, 0.6, 0.8, and 1.0 mg/mL of samples were prepared. The ABTS^+^ solution was prepared by mixing 5 mmol/L ABTS^+^ absolute ethanol and 2.1 mmol/L K_2_S_2_O_4_ solution in the dark for 16 h. The ABTS^+^ solution was diluted with absolute ethanol to reach an absorption rate of 0.70 ± 0.02 cm^−1^. In total, 2 mg of the sample was mixed with ABTS^+^ 1 mL (test group); 2 mg of the sample was mixed with 1 mL of absolute alcohol; and 2 mL of deionized water and 1 mL of ABTS^+^ solution were mixed. All mixtures were protected from light at room temperature, and the reaction was carried out after 30 min. Next, the absorbance was measured at 730 nm. Rg5, β-CD, β-CD-Rg5, and the β-CD/Rg5 physical mixtures were prepared in the same way. The reference standard was Vitamin C. The following equation was used:

$$ABTS+radical\;scavenging\;activity\;\left(\%\right)\;=1-\frac{A0-A1}{A2}\times 100$$

where A0 is the absorption of the test group, A1 is the absorption of the control group, and A2 is the absorption of the blank group. All samples were tested three times. 

#### 2.7.3. Fe^2+^ Chelating Ability

Determination of the Fe^2+^-chelating capacity of Rg5, β-CD, the β-CD-Rg5 inclusion complex, and the physical mixture was carried out as previously described by Li et al. [25]. Briefly, 0.2, 0.4, 0.6, 0.8, and 1.0 mg/mL of samples were prepared. Next, 10 µL of FeCl_2_ (2 mM) solution was added to 200 µL of the sample. After 15 min, 20 µL of 5 mM ferrozine was added, and kept at room temperature for 5 min. The samples were centrifuged for 10 min at 5000 rpm, and the absorbance of the supernatant solution was measured at 560 nm. The same procedure was followed for the Rg5, β-CD, β-CD-Rg5, and β-CD/Rg5 physical mixtures. EDTA-2Na was a reference standard. The following equation was used:

$$Fe2+Chelating\;ability\;(\%)=1-\frac{A0-A1}{A2}\times 100$$

where A0 is the absorption of the test group, A1 is the absorption of the control group, and A2 is the absorption of the blank group. All samples were tested three times.

### 2.8. Statistical Analysis

The resultant data are represented as the mean ± SD. All data were analyzed using a one-way ANOVA and Tukey’s multiple comparisons test. A significant difference between different groups was declared at a *p* value of 0.05. 

## 3. Results and Discussion

### 3.1. Effect of Amino Acids on Rg5 Conversion Rates in GSLS

An Acquity UPLC (UPLC) (Manchester, UK; Waters, Wilmslow, UK) with an Acquity UPLC R BEHC18 UPLC column (2.1 mm × 50 mm, 1.7 µm) was used. Water and acetonitrile were used as the mobile phase and a gradient elution process from 0 to 6.8 min (16–25% B), 6.8 to 20.5 min (25–40% B), 20.5 to 30.0 min (40–55% B), 30.0 to 35.5 min (55–65% B), and 35.5 to 40 min (65–16% B). The flow rate was 0.4 mL/min. The UV detection wavelength was set at 203 nm.

**Figure 2:**
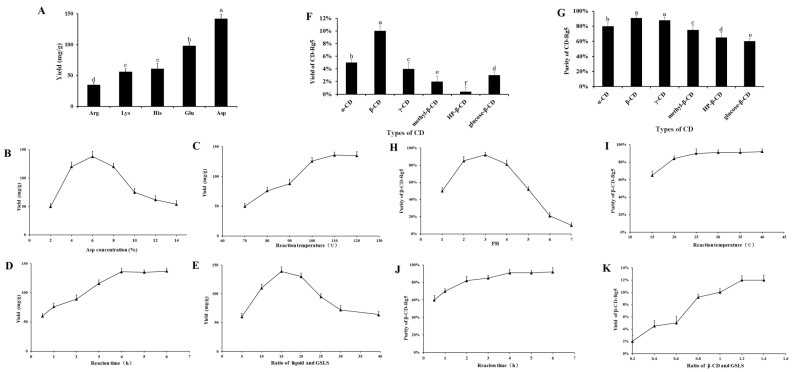
Effect of different conditions on the conversion contents of Rg5: (**A**) amino acids, different letters mean significantly different amounts (*p* < 0.05); (**B**) Asp concentration; (**C**) temperature; (**D**) reaction time; (**E**) liquid–solid ratio; (**F**,**G**) effects on yield and purity of β-CD-Rg5 inclusion complex—different letters mean significantly different amounts (*p* < 0.05); (**H**) pH of reaction system on purity of β-CD-Rg5 inclusion complex; (**I**) reaction temperature on purity of β-CD-Rg5 inclusion complex; (**J**) reaction time on purity of β-CD-Rg5 inclusion complex; (**K**) ratio of β-CD and DSLS on yield of β-CD-Rg5 inclusion complex.

The effect of different amino acids on Rg5 conversion is shown in Figure 2A. Five amino acids had obvious effects on the transformation of Rg5. The transformation effects of Arg, Lys, His, Glu, and Asp on Rg5 successively increased. The conversion rates of Rg5 using acidic amino acids were more efficient than those using basic amino acids. The content of Rg5 was 130 ± 2.17 mg/g when Asp was used. The conversion rate of Rg5 was significantly higher than that of strong inorganic acids; therefore, Asp was selected as the best catalyst.

The conversion of Rg5 was affected greatly by the concentration of amino acids. The results showed that when the concentration of Asp increased (2~6%), the conversion rate of Rg5 also increased, as shown in Figure 2B. When Asp was 6%, the highest conversion rate of Rg5 was achieved (138.5 ± 5.9 mg/g). Above 6%, the Rg5 conversion rate decreased with increasing amino acid concentration. The results showed that the optimal concentration of Asp was 6%. Temperature had a considerable influence on the formation of Rg5. The conversion rate of Rg5 gradually increased to some extent (Figure 2C). The conversion rate of Rg5 was highest at 100 °C, reaching 132.5 ± 5.6 mg/g; from the perspective of mass production, a temperature of 100 °C is easier to achieve, consumes less energy, and has a higher conversion efficiency. Therefore, the reaction temperature finally selected was 100 °C. The conversion rate of Rg5 reached a maximum within 4 h. Rg5 increased with the reaction time. The conversion rate of Rg5 was highest after 4 h (136.2 ± 4.3 mg/g). The conversion rate of Rg5 did not continue to increase beyond 4 h; this was due to the continuous consumption of the matrix during the reaction time. After the maximum active component is consumed, the effective collision decreases; thus, the content ultimately remains unchanged (see Figure 2D). The conversion rate of Rg5 first increased and then decreased with increasing liquid-solid ratio. At 15 mL/g, the conversion of Rg5 was highest at 139.5 ± 6.1 mg/g. The preferred liquid-to-solid ratio was 15 mL/g. The conversion of the liquid-solid ratio of Rg5 is shown in Figure 2E.

### 3.2. Optimization and Preparation of β-CD-Rg5 from GSLS

Natural CDs, α-CD, β-CD, and γ-CD, and other CD derivatives such as methyl-β-CD, HP-β-CD, and glucose-β-CD were used to improve physical properties and packaging compound properties. Figure 2F,G demonstrate the relative yield and purity of CD-Rg5, respectively, and were tested with six CDs. The results showed that the yield and purity of β-CD-Rg5 were 10% and 92%, respectively. The reason for this difference is that the compounds have different affinities to spaces and substituents. β-CD contains seven glucopyranose units and has a larger cavity to more easily accommodate Rg5, whereas α-CD and γ-CD contain six and eight units, respectively. As a result, spaces that are too large or too small are less likely to carry Rg5, which could account for the different extraction efficiencies. Three β-CD derivatives gave lower yields and purities of the CD-Rg5 inclusion complexes compared with those of β-CD, which could be because the inclusion of Rg5 is affected by the different chemically branched chains of CD derivatives. To obtain the optimal inclusion process of Rg5, the mass ratio of GSLS to β-CD, reaction time, reaction temperature and reaction solution pH were selected as factors for investigation (Figure 2H–K). With increasing reaction temperature, the purity of β-CD-Rg5 continuously increased. When the temperature was 30 °C, the purity of β-CD-Rg5 reached 92.5%; with increasing pH, the purity of β-CD-Rg5 increased. The purity of β-CD-Rg5 reached a maximum at pH = 3, and then decreased. The maximum purity was reached when the reaction time was 3 h; when the mass ratio of Rg5 to β-CD was 1:1.2, the yield of β-CD-Rg5 reached a maximum of 12%.

### 3.3. Characterization of β-CD-Rg5

#### 3.3.1. Confirmation of the β-CD-Rg5 Inclusion Complex Using ESI-MS

To prevent the destruction of noncovalent bonds in the gas phase, the ESI-MS parameters were optimized. The optimal experimental conditions were a 20 psi air curtain; potential energy and collision energy of 20 V and 20 eV, respectively; an ion injection voltage of 3000 V; a nozzle gas at 30 psi; and a declustering voltage of 80 V. The scanning speed was 1000 mu s^−1^. All samples were injected at a rate of 5 μL min^−1^ with a 3.7 mm diameter syringe pump. In the appropriate mass range, each sample was acquired for 2 min.

**Figure 3:**
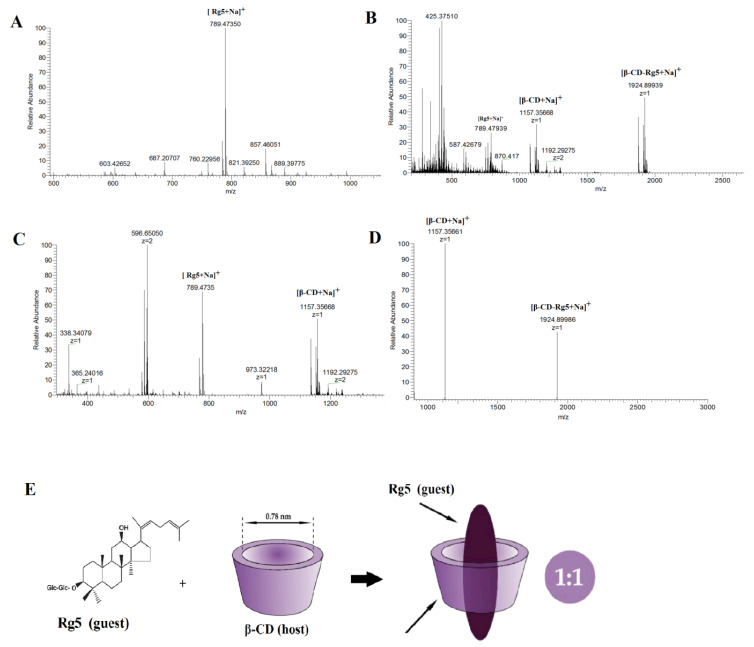
Positive ion ESI mass spectrum for Rg5 (**A**); the β-CD-Rg5 inclusion complex (**B**); the β-CD/Rg5 physical mixture (**C**) and ESI tandem mass spectrum of [β-CD-Rg5+Na]^+^ at *m*/*z* 1924.89939 (**D**); host–guest complex stoichiometry of β-CD and Rg5 (**E**).

Single, binary, ternary, and multiple inclusion complexes can be formed between CD and guest molecules. Stoichiometric ratios of inclusion complexes can typically be obtained via the equimolar continuous change method and the Benesi–Hildebrand equation. The most popular method is to use mass spectrometry to provide stoichiometric information [26]. The results showed that compared with the negative ion mode, β-CD easily forms an excimer ion peak with Na^+^ in the positive ion mode, and the mass-to-charge ratio *m*/*z* 1157.5 is [β-CD +Na]^+^. Rg5 tends to form an excimer ion peak with Na^+^, and the mass to charge ratio *m*/*z* 789.47350 is [Rg5+Na]^+^. Figure 3A shows the mass spectrometry with a β-CD and Rg5 physical mixture in positive ion mode and the *m*/*z* 1157.5 is [β-CD+Na]^+^ and the *m*/*z* 789.4735 is [Rg5+Na]^+^. The β-CD-Rg5 inclusion complex produces a stronger signal in the positive ion mode. Figure 3B shows the mass spectrometry of the β-CD-Rg5 inclusion complex in positive ion mode: *m*/*z* 1924.89939 is [β-CD-Rg5+Na]^+^, *m*/*z* 1157.5 is [β-CD+Na]^+^, and there is no [Rg5+Na]^+^. Further analysis by tandem mass spectrometry showed that the fragment of the inclusion complex [β-CD-Rg5+Na]^+^ *m*/*z* 1924.89939 was broken up to produce [β-CD+Na]^+^ *m*/*z* 1157.35661 (Figure 3C), indicating that the neutral molecule of Rg5 was lost in the β-CD-Rg5 inclusion complex. This indicated that β-CD formed a 1:1 complex with Rg5, which can be seen in Figure 3D,E.

#### 3.3.2. FTIR Spectroscopy

**Figure 4:**
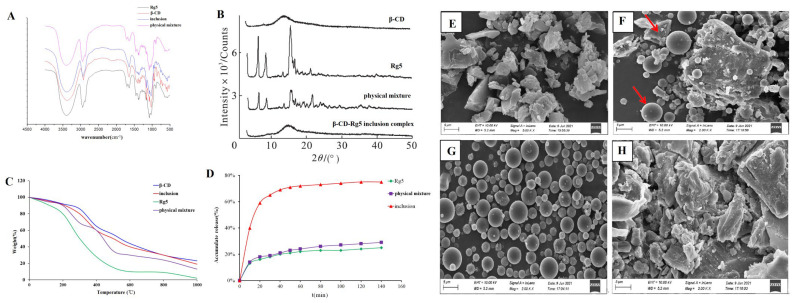
(**A**) FTIR and (**B**) XRD spectrum. (**C**) TGA thermograms. (**D**) Dissolution curve of Rg5, β-CD, β-CD-Rg5 inclusion complex and the β-CD/Rg5 physical mixture. SEM analysis of β-CD (**E**); the β-CD/Rg5 physical mixture (**F**); Rg5 (**G**); the β-CD-Rg5 inclusion complex (**H**). Red arrow means the shape of β-CD and Rg5.

Rg5, β-CD, the β-CD-Rg5 inclusion complex, and the physical mixture were taken, the tablets were pressed with potassium bromide, and the IR spectra were determined. As shown in Figure 4A, the characteristic absorption peaks of Rg5 were associated with hydroxyl groups (3374.5 cm^−1^ and 3277.4 cm^−1^), submethyl groups (2945.2 cm^−1^) and carbonyl groups (1387.2 cm^−1^). The characteristic absorption peaks of β-CD were hydroxyl (3412.7 cm^−1^), methyl (2966.9 cm^−1^), submethyl (2927.3 cm^−1^), carbonyl (1638.4 cm^−1^), etc. The spectra of the physical mixture showed the superposition of Rg5 and β-CD absorption spectra, whereas the spectra of the inclusion complex were similar to those of β-CD. The characteristic peak of Rg5 in the inclusion complex was covered by the characteristic peak of β-CD, indicating that an inclusion complex had been formed. At the same time, no new absorption peak appeared in the scanning range of the inclusion complex, indicating that no covalent bond was formed between the two during the formation of the inclusion complex, and the combination was in the form of noncovalent bonds.

#### 3.3.3. X-ray Diffraction Analysis

The results showed that Rg5 had a very strong crystal diffraction peak (Figure 4B). The diffraction peaks of Rg5 and β-CD were superimposed in the physical mixture, but the intensity of the peak was slightly changed. Rg5 still existed as a crystal in the system. The spectra of the inclusion complex showed that the crystal diffraction peak of Rg5 disappeared, which was generally consistent with the blank carrier, indicating that Rg5 was highly dispersed in the carrier in an amorphous state, which was conducive to the dissolution of Rg5.

#### 3.3.4. Thermogravimetric Analysis

Under controlled conditions, thermogravimetric analysis was used to determine the mass loss of the samples (Figure 4C). TG analyses were performed on Rg5, β-CD, β-CD-Rg5, and β-CD/Rg5 physical mixtures, and their thermal properties were compared. For Rg5, its mass gradually decreases from room temperature to 400 °C. However, the quality dropped drastically until almost no sample was maintained at 400 °C or higher (0%). This shows that the thermal decomposition of Rg5 at 300~400 °C is complete. Samples containing β-CD showed similar responses during thermal decomposition. When the ambient temperature reached 200 °C, the quality decreased gradually, and when it reached 200 °C to 600 °C, the quality decreased rapidly and then gradually decreased from 600 °C to 800 °C. However, a large amount of sample remained after heating. For example, the original masses of β-CD, the β-CD-Rg5 inclusion complex and the β-CD/Rg5 physical mixture remained at approximately 35%, 25% and 20%, respectively, after heating. Because the β-CD-Rg5 inclusion complex increases the thermal stability of Rg5, the mass loss of the β-CD-Rg5 inclusion complex is smaller than those of Rg5 and the β-CD/Rg5 physical mixture.

#### 3.3.5. SEM

The results showed that Rg5 was a spherical crystal and formed an agglomerated state (Figure 4E–H). β-CD appeared as an irregular crystal; the physical mixture took the form of CD and Rg5 separately with some aggregates of Rg5 molecules around the β-CD. In contrast, the morphology and crystal size of the inclusion complex are completely different to those of the original forms of β-CD, Rg5, and the physical mixture, indicating that the two molecules interact with each other and further demonstrating that β-CD and Rg5 form the inclusion complex.

### 3.4. Stability of β-CD-Rg5

**Figure 5:**
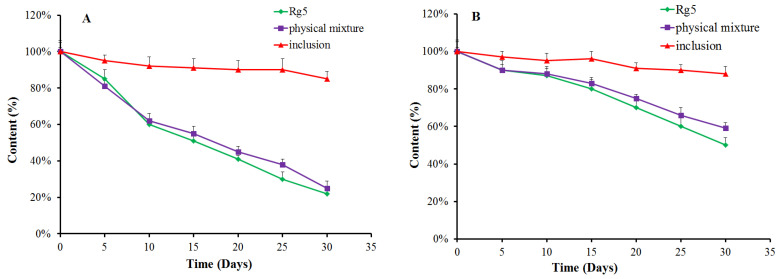
Antioxidant retention (%) of Rg5, the β-CD-Rg5 inclusion complex and the β-CD/Rg5 physical mixture exposure to light (**A**) and at 45 °C (**B**).

We evaluated the stability of Rg5, the β-CD-Rg5 inclusion complex and the physical mixture exposed to light (Figure 5A) and a temperature of 45 °C (Figure 5B). The results showed that Rg5 exposed to light and temperature was unstable. The amount of Rg5 appeared to markedly decrease when subjected to light treatment for 30 d. Interestingly, the β-CD-Rg5 inclusion complex effectively retarded Rg5 degradation. Rg5 achieved the fastest degradation rate at high temperature within 1 h. The degradation rates of the β-CD-Rg5 inclusion complex were significantly retarded at 45 °C. The results strongly supported that the grafting of Rg5 to the β-CD-Rg5 inclusion complex was an effective approach for improving stability.

### 3.5. Dissolution In Vitro

After natural ingredients are complexed with CD, the solubility of the inclusion complex in water is generally improved to varying degrees compared with that before inclusion, particularly for substances that are insoluble in water [27]. The dissolution rate of Rg5 in water is greatly slow, and the rate of dissolution tends to plateau after 20 min, as shown in Figure 4D. The β-CD-Rg5 inclusion complex dissolved rapidly in the first 20 min, and then the dissolution rate decreased and tended to stabilize. The cumulative dissolution of the inclusion complex reached 78.5% at 70 min, and the dissolution rate was significantly higher than those of Rg5 and the physical mixture; the solubilization effect of the β-CD-Rg5 inclusion complex was up to 3.8 times greater.

### 3.6. Antioxidant Activity

**Figure 6:**
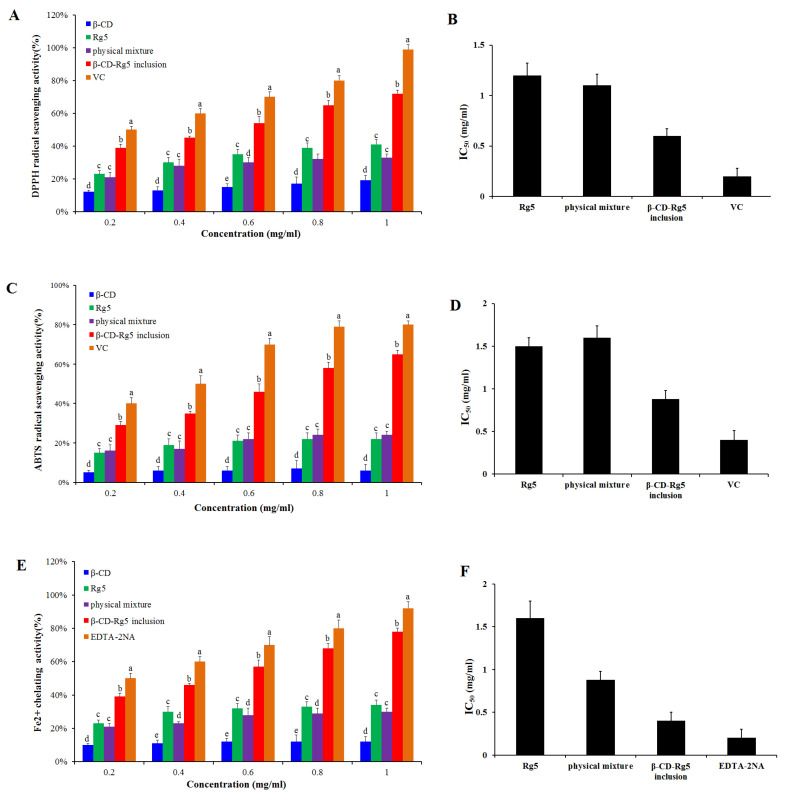
DPPH radical scavenging ability (**A**) and IC50 (**B**); ABTS^+^ radical scavenging ability (**C**) and IC50 (**D**); Fe^2+^ chelating ability (**E**) and IC50 (**F**) of Rg5, the β-CD-Rg5 inclusion complex and the β-CD/Rg5 physical mixture exposure. Different letters mean significantly different (*p* < 0.05).

Antioxidant activity was determined for the β-CD-Rg5 inclusion complex. Various samples were compared via DPPH scavenging, ABTS^+^ scavenging and Fe^2+^ chelating abilities. Figure 6A,B showed that when the concentration of Rg5, the β-CD-Rg5 inclusion complex, the β-CD/Rg5 physical mixture and vitamin C increased, the clearance rate of DPPH increased and showed concentration dependence. In the samples, the respective scavenging efficiencies of DPPH were 40.1%, 38.5%, 70.5%, and 95.5%. The results showed that the half-inhibitory concentrations of Rg5, the physical mixture, and the β-CD-Rg5 inclusion complex were 1.22 mg/mL, 1.13 mg/mL and 0.61 mg/mL, respectively; the antioxidant capacity of each sample was enhanced with increasing concentrations. However, the results showed that the antioxidant capacity of each test sample was lower than that of vitamin C. Compared with pure Rg5 (IC50 = 1.22 mg/mL), the antioxidant activity of DPPH was enhanced in the presence of β-CD (IC50 = 0.61 mg/mL), which was due to the inclusion of Rg5 in β-CD releasing more hydrogen atoms bound to DPPH radicals in water, leading to the reduction of DPPH radicals [28]. A previous research report on the DPPH radical scavenging activity of curcumin and its complex showed that CD inclusion complexes can enhance the antioxidant capacity of curcumin [29]. The inclusion complex of β-CD-Rg5 (IC50 = 0.61 mg/mL) was significantly higher (*p* < 0.05) than that of Rg5 (IC50 = 1.22 mg/mL). Figure 6C,D show that the β-CD-Rg5 inclusion complex has a stronger ABTS^+^ clearance capacity (IC50 = 1.51 mg/mL) than Rg5 (IC50 = 1.51 mg/mL). Compared with the DPPH value, the two are consistent. The reaction principles of the two detection methods used in this study differ. The DPPH method based on hydrogen atom transfer quenches the free group by detecting the ability of the antioxidant agent to provide a hydrogen ion [30]. ABTS^+^ reduces the oxidation of free radicals by detecting the transfer of one electron by antioxidants [31]. The two types of determination methods complement each other and can fully evaluate the resistance oxidation capacity of Rg5 before and after inclusion.

In free radical oxidation, metal ions act as catalysts. Metal complexes can effectively inhibit the catalytic activity of metal ions, thereby preventing the generation of free radicals. Therefore, it is good practice to measure the activity of metal complexes. Ferrozine can form a purple chelate with Fe^2+^. If other competing chelating agents are present, the color will be lighter. Thus, based on the color change of the complex, the complexing capacity of iron ions can be judged. Figure 6E,F show that the Fe^2+^ chelation capacity is improved with increasing concentration of each sample. However, in each case, the tested samples had a lower chelating capacity than EDTA-2Na (IC50 = 0.19 mg/mL). The chelating ability of Rg5 (IC50 = 1.5 mg/mL) is higher than that of β-CD-Rg5 (IC50 = 0.88 mg/mL). Studies have found that the β-CD-Rg5 inclusion complex can improve the antioxidant capacity of Rg5 and can indirectly inhibit the generation of hydroxyl radicals through complexation [25].

## 4. Conclusions

In this study, a novel and effective strategy for the preparation of the β-CD-Rg5 inclusion complex from GSLS was developed. The isolation and inclusion of Rg5 with β-CD were achieved in one step. The common ginsenosides in GSLS were converted into Rg5 using Asp as a catalyst. The content of Rg5 reached 140.8 mg/g after transformation of GSLS. Different CDs and reaction conditions were investigated for the preparation of CD-Rg5. The yield of β-CD-Rg5 reached a maximum of 12% and a purity of 92.5%. The results of ESI-MS, SEM, XRD, and FT-IR analyses proved the formation of the β-CD-Rg5 inclusion complex. The β-CD-Rg5 inclusion complex improves its storage stability and increases the solubility of Rg5 from 25.5% to 78.5%. This greatly enhances the antioxidant activity of Rg5. This work promotes the industrial production of Rg5 and its application in functional foods.

## Data Availability

The data used to support the findings of this study can be made available by the corresponding author upon request.
